# Runs of homozygosity analysis reveals consensus homozygous regions affecting production traits in Chinese Simmental beef cattle

**DOI:** 10.1186/s12864-021-07992-6

**Published:** 2021-09-21

**Authors:** Guoyao Zhao, Yuqiang Liu, Qunhao Niu, Xu Zheng, Tianliu Zhang, Zezhao Wang, Lei Xu, Bo Zhu, Xue Gao, Lupei Zhang, Huijiang Gao, Junya Li, Lingyang Xu

**Affiliations:** Key Laboratory of Animal Genetics Breeding and Reproduction, Ministry of Agriculture and Rural Affairs, Institute of Animal Science, Chinese Academy of Agricultural Sciences, Yuanmingyuan West Road 2#, Haidian District, 100193 Beijing, China

**Keywords:** Runs of homozygosity, Production traits, Consensus ROH, Association analysis, Chinese Simmental beef cattle

## Abstract

**Background:**

Genomic regions with a high frequency of runs of homozygosity (ROH) are related to important traits in farm animals. We carried out a comprehensive analysis of ROH and evaluated their association with production traits using the BovineHD (770 K) SNP array in Chinese Simmental beef cattle.

**Results:**

We detected a total of 116,953 homozygous segments with 2.47Gb across the genome in the studied population. The average number of ROH per individual was 99.03 and the average length was 117.29 Mb. Notably, we detected 42 regions with a frequency of more than 0.2. We obtained 17 candidate genes related to body size, meat quality, and reproductive traits. Furthermore, using Fisher’s exact test, we found 101 regions were associated with production traits by comparing high groups with low groups in terms of production traits. Of those, we identified several significant regions for production traits (P < 0.05) by association analysis, within which candidate genes including *ECT2*, *GABRA4*, and *GABRB1* have been previously reported for those traits in beef cattle.

**Conclusions:**

Our study explored ROH patterns and their potential associations with production traits in beef cattle. These results may help to better understand the association between production traits and genome homozygosity and offer valuable insights into managing inbreeding by designing reasonable breeding programs in farm animals.

**Supplementary Information:**

The online version contains supplementary material available at 10.1186/s12864-021-07992-6.

## Background

Runs of homozygosity (ROH) is defined as contiguous regions of the genome where an individual is homozygous across sites. ROH arises when the haplotypes transmitted from the parents are identical and inherited from a common ancestor [1]. Increasing the proportion of homozygous loci and generating homozygous segments can be used to reflect the loss of genetic diversity [2] and the performance of traits in livestock [3].

Many studies revealed the negative impact of high homozygosity on fertility traits including bull semen quality [4], calving rate [5], stillbirths, and dystocia [6]. Therefore, controlling inbreeding in modern breeding programs and maintaining genetic diversity have attracted major attention within breeding schemes [7, 8].

Previous studies have been carried out to explore ROH and its potential association with diseases in humans [9–12]. Investigation of ROH in farm animals also suggested their important contributions to complex traits [2]. For instance, a previous study evaluated the association between ROH and reproduction traits using whole-genome homozygosity mapping and revealed candidate regions affecting bull fertility in US Holstein cattle [13]. Moreover, a recent study identified ROH that unfavorably affects female fertility and milk production traits in the Finnish Ayrshire population [14].

Allocating ROH into different classes based on length can be used to separate recent and ancient inbreeding. Long ROH may reflect recent inbreeding with a very low probability of recombination, while short ROH may indicate ancient inbreeding [15]. Based on genetic purging theory, ancient inbreeding that occurred from a distant common ancestor is expected to show less unfavorable effect due to purging, whereas recent inbreeding arising from a most recent common ancestor may exhibit larger unfavorable effects [16]. Genome-wide inbreeding depression was observed for milk yield and udder health traits in dairy cattle. For instance, a previous study suggested that the increase in genome-wide homozygosity was associated with a decrease in milk yield [17]. In contrast, another study evaluated the effect of recent and ancient inbreeding on production and fertility traits, and their findings suggested recent genomic inbreeding showed more detrimental inbreeding effects in Canadian Holsteins, while more distant ancient inbreeding may cause favorable effects [18]. However, the relationship between ROH and economically important traits and the effect of recent and ancient inbreeding on production traits in beef cattle are still not fully explored.

The objectives of this study were to evaluate the homozygous segment pattern at the whole genome level in Chinese Simmental beef cattle and assess the association between ancient homozygous segments and production traits.

## Results

### Assessment of runs of homozygosity

**Fig. 1:**
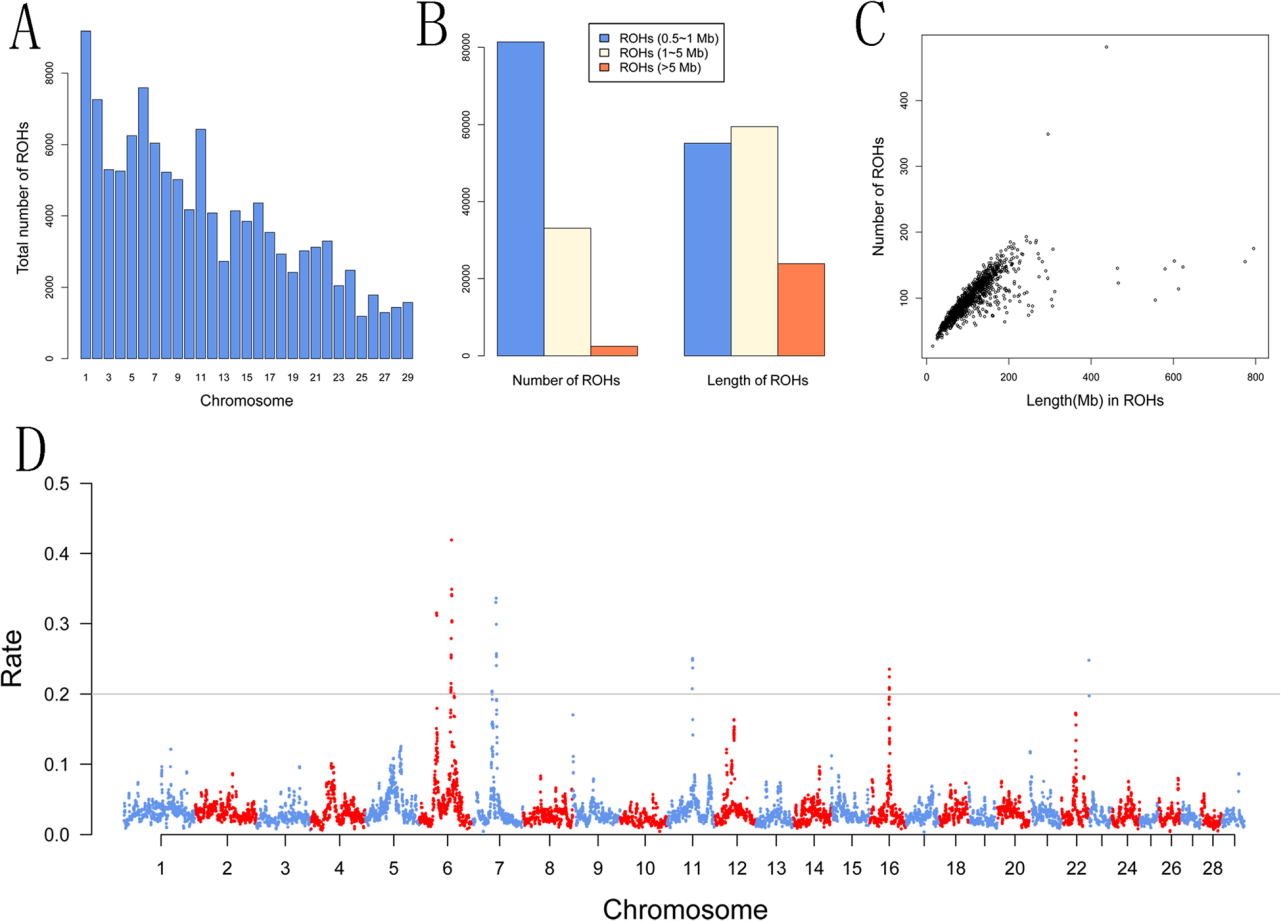
(**A**) The distribution of the total number of ROH across chromosomes. (**B**) The total number and length (Mb) of ROH belonging to three size classes including Small (0.5 to 1 Mb), Medium (1 to 5 Mb), and Large (> 5 Mb) size. (**C**) Evaluation of the number of ROH and ROH total length. The number of ROH found for each individual genome (y-axis) is plotted against ROH total length (i.e. the length of Mb covered by ROH in each genome, x-axis). (**D**) Distribution characteristics of ROH on chromosomes in Chinese Simmental beef cattle. The horizontal axis is the single nucleotide polymorphism (SNP) position, which is ordered by the physical location of the genome; the vertical axis is the ROH rate. The horizontal line in the graph is where the ROH frequency equals 20 %

In this study, we identified a total of 116,953 ROH with 2.47Gb across the genome in 1181 Chinese Simmental beef cattle. We found an average number of 99.03 ROH segments per individual with an average length of 117.29 Mb. Most ROH (~ 69.60 %) belong to the short class, ranging from 0.5 Mb to 1 Mb, while the large class only occupies a small portion (2.1 %) (Supplement Table S1). Moreover, we identified the largest ROH located on BTA9 (57,696.85 kb, with 13,143 SNPs), while the shortest ROH was observed on BTA7 (500.021 kb, with 123 SNPs). In addition, we observed that the number of ROH varied across autosomes (from 1,187 ROH on BTA25 to 9,176 ROH on BTA11). The distribution of the total number of ROH across chromosomes was presented in Fig. 1 A. The distribution of the total length of ROH across chromosomes was shown in (Supplement Figure S1). We found that the number of ROH per chromosome generally reflect the chromosome size.

We divided ROH into three classes: (A) Small (500 kb to 1 Mb), (B) Medium (1 Mb to 5 Mb), and (C) Large (> 5 Mb) [19]. The ROH distributions of total number and length for each class were presented in Fig. 1B. The proportion of ROH covering the genome with different lengths (thus inferred to be autozygous) varied in our population. Our result showed that the total length of Medium ROH was larger than Small and Large ROH. Small ROH was found to be predominant in the studied population by their total numbers. Moreover, we observed the total number of ROH and length of ROH were highly correlated (r = 0.69) (Fig. 1 C). Notably, extremely high correlations were found between the total lengths and total number for Small, Medium, and Large ROH (r = 0.996, 0.978, 0.963) (Supplement Figure S2).

### The consensus of ROH across the population

To investigate the distribution of ROH enrichment within the population, we carried out an analysis of consensus ROH using PLINK with –homozyg-group option. We observed the highest frequency of consensus ROH (> 42 %) was located in the middle part of BTA6. Two ROH regions with frequencies of 34 % and 25 % were located on BTA7 and BTA11, respectively. We also detected 14, 6, and 1 consensus ROH (showing a rate of more than 25 %) on BTA6, BTA7, and BTA11 respectively. The genome-wide plot of the distribution of ROH enrichment was shown in Fig. 1 D. In the present study, we totally detected 42 regions with the ROH rate exceeding 20 %, accounting for 17 RefGenes based on UMD 3.1. These genes include *DNAJC18, ECSCR, MATR3, PAIP2, SNHG4, SPATA24, TMEM173, UBE2D2, KED, DCAF16, LCORL, NCAPG, etc.* Moreover, we found several quantitative trait locus (QTLs) for weight gain and calving index overlapped with these regions based on the cattle QTLdb (https://www.animalgenome.org/cgi-bin/QTLdb/BT/index).

### Evaluation of correlation between ROH and production traits

**Fig. 2:**
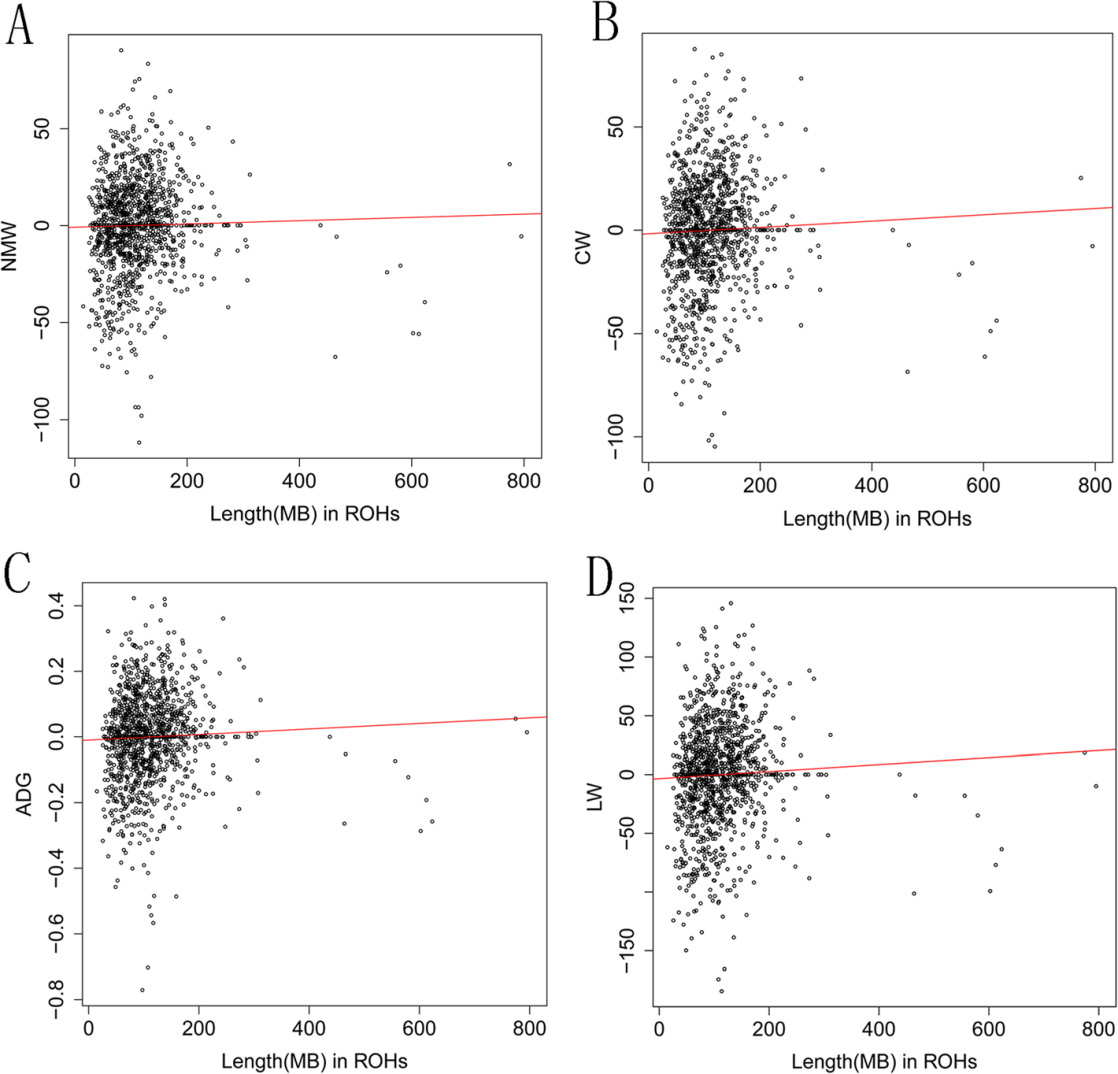
Scatter plot of total homozygosity per individual against four corrected carcass traits (NMW, CW, ADG, LW). (**A**) Net meat weight (NMW), (**B**) Carcass weight (CW), (**C**) Average daily gain (ADG); (**D**) Live weight (LW)

To assess the association between homozygosity level and production traits, we performed the correlation analyses between the total length of ROH for each animal and adjusted phenotype including net meat weight (NMW), carcass weight (CW), average daily gain (ADG), and live weight (LW). Notably, we found the Pearson’s correlation coefficient between the sum of ROH and four traits (NMW, CW, ADG, and LW) were 0.02, 0.04, 0.04, and 0.05(Fig. 2), respectively, whereas no significant difference was observed. Notably, we observed Pearson’s correlation coefficient between small ROH and four traits (NMW, CW, ADG, and LW) were 0.13, 0.15, 0.14, 0.15, while negative correlation coefficients were found when considering large ROH (-0.06, -0.054, -0.046, -0.049) (Supplement Figure S3, S4 and S5).

### Identification of ROH between high and low groups using Fisher’s exact test

In this study, a total of 5,305 consentient ROH were detected across the genome in Chinese Simmental beef cattle (Supplement Table S2). The average length of overlapping ROH was 101.5 kb, with an average of 26 SNPs. The largest region was 1,091 kb containing 20 SNPs, whereas the shortest one spanned 2.8 kb containing 5 SNPs.

**Fig. 3:**
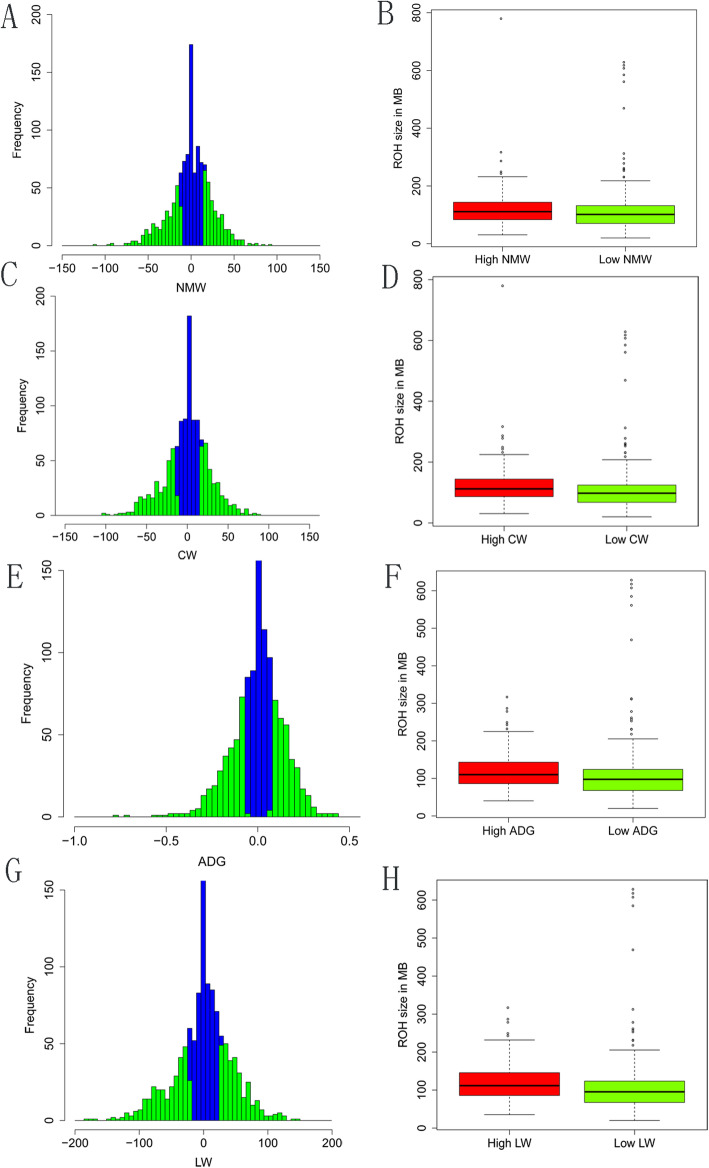
Correlation between runs of homozygosity and four carcass traits (NMW, CW, ADG, LW). (**A**) Histogram showing the distribution of NMW for Chinese Simmental beef cattle. (**B**) Distribution of total homozygosity, calculated as the sum of all runs of homozygosity in the high group and low group for NMW. (**C**). Histogram showing the distribution of CW. (**D**). Distribution of total of runs of homozygosity in the high group and low for CW. (**E**). Histogram showing the distribution of ADG. (**F**). Distribution of total of runs of homozygosity in the high group and low group for ADG. (**G**). Histogram showing the distribution of LW. (**H**). Distribution of total of runs of homozygosity in the high group and low group for LW

The level of homozygosity may reflect potential variation in production traits among individuals. We evaluated the ROH patterns in terms of total length between the 300 high and low groups (Fig. 3). We found the total length of ROH was significantly different between high and low groups for production traits using Wilcoxon Rank Sum Tests (CW, P = 1.85e-05; LW, P = 3.25e-06; NMW, P = 1.56e-03; ADG, P = 5.05e-05). Then, the enrichments of consensus ROH were assessed using Fisher’s exact test by comparing the top 300 high groups against the bottom 300 low groups. The consensus ROH were filtered and only those containing at least five SNPs were considered for subsequent analysis. Finally, 36, 35, 43, and 50 regions were obtained for NMW, CW, ADG, and LW.

### Association analysis of significant ROH

To further validate the association between ROH and production traits, we performed association analysis using a mixed linear model as proposed by previous studies[13]. After the Fisher’s exact tests, we found 36, 35, 43, and 50 candidate ROH regions for NMW, CW, ADG, and LW, respectively. Of these regions, 7 regions were significantly associated with production traits (P-value < 0.05). The summary statistics of candidate ROH regions and their candidate genes were listed in (Supplement Table S3).

**Fig. 4:**
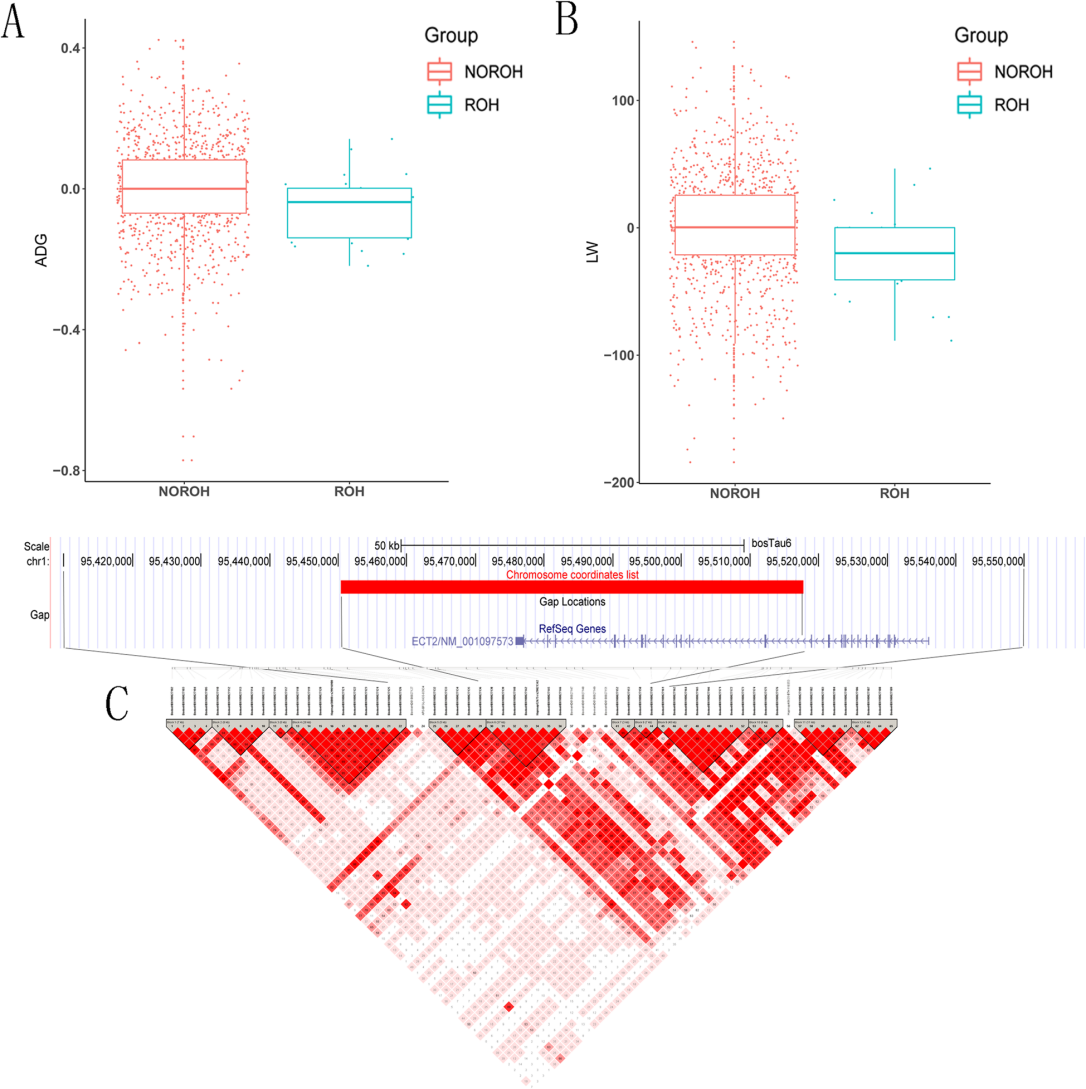
(**A**). The boxplot of the adjusted ADG for ROH and non ROH; (**B**). The boxplot of the pre-adjusted LW for ROH and non ROH; (**C**). Haplotype LD analyses nearby the ROH (chr1:95,450,372–95,517,810) on candidate gene *ECT2*

After removing the redundant regions (as several regions were identified for two production traits), we obtained four unique candidate regions. Totally, we observed four genes related to production traits, which may play an important role in promoting growth and metabolic functions. These candidate genes were found in the ROH regions located on BTA1, BTA5, BTA6, and BTA9. Of these, we found one region located at (chr1:95,450,372–95,517,810) for average daily gain and live weight, which was largely overlapped with *ECT2*. Moreover, significant differences were observed for adjusted ADG (P = 7.92 × 10^− 3^) and LW (P = 2.26 × 10^− 3^) between individuals with ROH and those without ROH (Fig. 4 A and B).

Consensus ROH that occur prevalently throughout the genome due to LD [20]. Our findings are consistent with a previous study by Purfield et al. which revealed that a majority of SNPs in the ROH region were in high LD with nearby SNPs [21]. Notably, we observed strong LD existed within the shared region between the ROH region and *ECT2* (Fig. 4 C). We also observed one ROH region at BTA6 (chr6:67,192,462–67,290,024) with three genes (*GABRA4*, *GABRB1*, *LOC536190*) associated with CW (P = 0.0110) and NMW (P = 0.0052). However, no significant differences were observed for adjusted CW and NMW between individuals with ROH and those without ROH. In addition, no gene was detected in two candidate regions at BTA5 and BTA9.

## Discussion

The increasing use of key sires has been coupled with a drastic reduction in the generation interval for most cattle populations, representing both an opportunity and a challenge [22]. Artificial insemination may lead to the rapidly accumulating of homozygous segments. In the present study, we attempted to study the relationship between genome homozygous regions and production traits in beef cattle and explored the potential candidate genes. We evaluated the relationship between ROH and production traits in Chinese Simmental beef cattle using the BovineHD 770 K SNP arrays. Secondly, we divided our population into high and low trait groups to explore the enrichment of ROH fragments in production traits. Finally, we identified candidate ROH regions in the cattle genome and  obtained several candidate genes that may affect production traits.

### Assessment of runs of homozygosity

In this study, we characterized homozygosity regions for the first time in Chinese Simmental cattle using a high-density SNP array. We found a large proportion of small ROH (~ 69.6 %), which was similar to our previous report [23]. The high-density SNP array is more sensitive to the determination of small segments [24], while the low-density array may underestimate the small ROH. Many studies have reported that long ROH are mostly enriched in deleterious mutation regions, and inbreeding can increase the occurrence of rare recessive diseases that are homozygous for deleterious mutations [25]. Recent inbreeding is expected to be more harmful than ancient inbreeding because selection reduces the frequency of deleterious alleles over generations [26]. Our study revealed that the average number of short ROH segments per animal is large, which implied that most of the ROH segments in  the studied population were derived from distant ancestors [19, 27].

### Identification of the high-frequency ROH among the population

**Fig. 5:**
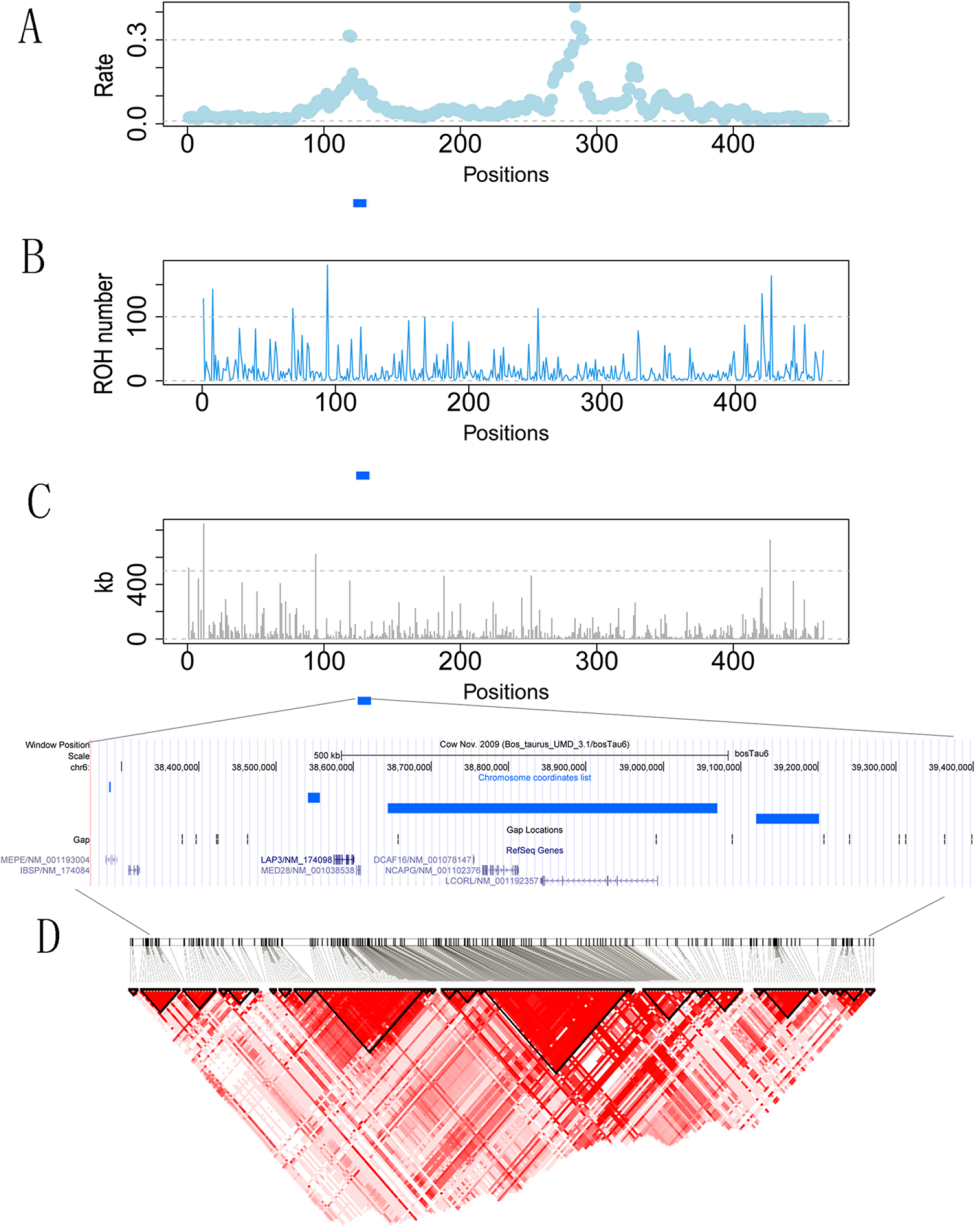
(**A**). The rate of ROH plot at BTA6. (**B**). The number of ROH based on the region extracted from BTA6. (**C**). The length of ROH is based on the region extracted from BTA6. (**D**). Haplotype LD analyses nearby the 1.2 Mb region with three consensus ROH were overlapped with three genes (*LAP3*, *NCAPG*, and *LCOLR*)

The selection of superior animals can yield large phenotypic changes and reshap the ROH patterns in specific regions across the genome. Previous studies have identified the most homozygous region (> 45 % of individuals with ROH) on BTA6 within QTLs affecting milk fat and protein concentrations in local dairy cattle breeds [28]. Our study identified several high-frequency regions with consensus ROH which were overlapped with QTLs for important traits in cattle including body weight, strength and rump width, average daily gain, and shear force. For instance, we observed several peaks representing the high-frequency ROH region on BTA6 (Fig. 5 A), while most of the ROH were less than 400 kb (Fig. 5 B and C).

Notably, the small ROH are mainly selected and derived from ancient haplotypes [29]. Therefore, these ROH are likely to be undergoing positive selection for important traits and thus they were enriched with high frequency in the population. Remarkably, we observed a 1.2 Mb region on BTA6 with three consensus ROH, which was overlapped with genes (*LAP3*, *NCAPG*, and *LCORL*). Of those, we identified the largest ROH (425.68 kb) embedded with both *NCAPG* and *LCORL*. These two genes have been indicated that were related to growth and carcass merit traits in many previous studies [30]. Moreover, many candidate variants were detected within or nearby *NCAPG* and *LCORL* using multiple strategies in various cattle breeds [31–35]. These two genes have also been implied to undergone positive selection in cattle and other farm animals [36–41]. Remarkably, our study revealed a high LD pattern around this region which may indicate potential selection for favor allele and extend LD around them (Fig. 5D).

### Correlation between ROH characteristics and production traits

To assess the effect of homozygosity on the production traits, we estimated the relationship between the total length of ROH for each individual and the production traits (NMW, CW, ADG, and LW). Our study revealed the total length of ROH in autosomes across individuals was weakly correlated with production traits, but not statistically significant.

Negative effects of inbreeding depression was observed on production traits in various dairy cattle [42–45]. Despite many previous studies suggested a negative association between bull fertility and the amount of homozygosity by assessing the total homozygous regions for each animal (sum of ROH) versus sire conception rate records [13]. Conversely, an increase in performance also occurs for ancient inbreeding that arose from a distant common ancestor. For instance, Doekes et al. [26] reported an increase of 0.03 kg for ancient inbreeding in Dutch Holstein-Friesian dairy cattle. Moreover, a recent study suggested that a 1.33 kg gain for 305-day protein yield was found per 1 % increase in short ROH, representing the most distant pedigree age class in Canadian Holsteins [18].

Our study showed that the inbreeding coefficient of the Chinese Simmental beef cattle estimated from ROH is around 0.047, suggesting the population has not experienced inbreeding. In our study, we observed an abundance of short ROH across the genome in the studied population. The short ROH derived from ancient haplotype which may have favorable inbreeding effects as compared with recent age of inbreeding. This finding may support the previous findings that ancient inbreeding occurred from a distant common ancestor is expected to display less unfavorable effects due to genetic purging [16, 26].

### Identification of candidate genes for production traits based on ROH analysis

We compared the distribution of ROHs for high and low production trait groups (300 top and 300 bottom individuals). Notably, a clear distribution difference was observed between the two groups in terms of the total length of ROH, which implied the difference in the levels of homozygosity between the two groups. Notably, we identified 101 nonredundant regions associated with production traits using Fisher’s exact test by comparing the top 300 high groups against the bottom 300 low groups. These regions account for a total of 120 candidate genes. Of those genes, several genes with candidate regions were observed that showing potential associations with reproduction traits as reported in previous publications. For instance, the region on BTA9 was identified for LW, NMW, ADG, and CW harboring three candidate genes (*FAM229B, LAMA4*, and *TUBE1*). *LAMA4* was localized and upregulated in damaged muscle fibers, and this gene appears to contribute to fiber survival in zebrafish [46]. Moreover, *LAMA4*-/- mice were reported that exhibited reduced weight gain in response to both age and high-fat diet [47]. This gene has been indicated that closest to candidate QTL with pleiotropic effect on body composition [48]. In addition, several studies also suggested that *LAMA4* was a candidate gene for meat quality [49, 50].

To further provide the association evidence, we performed an association test based on the identified ROH using a mixed linear model. Importantly, *ECT2* was previously identified for live weight and average daily gain. A recent study using weighted single-step association reported one region explains more than 0.5 % of the additive genetic variance for residual intake and body weight gain overlapped with *ECT2* in Nellore cattle [63].

Moreover, *GABRA4* and *GABRB1* belonged to gamma-aminobutyric acid (GABA)ergic Synapse, have been previously reported related to reproduction trait [51]. These genes involved in GABAergic signaling were included in the response to the organic cyclic compound biological process, which was related to conformation score [52]. GABA is synthesized from glutamate by the enzyme glutamic acid decarboxylase and was reported to play a role in controlling feeding behavior in ruminant animals. The GABAergic synapse pathway has also recently been associated with live weight in Simmental cattle [53]. In addition, *GABRB1* and *GABRA4* were reported as candidate genes affecting meat color traits in Nellore cattle [54].

## Conclusions

We characterized the ROH and evaluated the association between ROH and production traits in Chinese Simmental beef cattle. Our study suggested that consensus ROHs derived from ancient haplotype may have a positive impact on production traits in beef cattle. Our findings provided a better understanding of the molecular basis underlying production traits from the aspect of homozygosity across cattle genomes.

## Methods

### Ethics statement

No ethics statement was required for the collection of genetic material. The data from animals included in this study were derived from previous analyses that obtained specific permissions.

### Phenotypic and genotypic data

The dataset includes the phenotypes of 1,181 Chinese Simmental beef cattle born between 2008 and 2015 from Ulgai, Xilingol League, and Inner Mongolia, China. Average Daily Gain (ADG) was obtained with body weight gain divided by the number of fattening days. Carcass’s merit traits were measured as described in our previous analysis [31, 55].

Samples were genotyped with Illumina BovineHD BeadChip and were processed with Genome Studio software. The individual call rate > 95 % was kept, and SNP quality controls were carried out using PLINK v1.9 [56] based on minor allele frequency (> 0.05), the proportion of missing genotypes (< 0.05), Hardy-Weinberg equilibrium (P > 10e-6). After quality control, 1181 individuals and 602,220 SNPs included in autosomes remained for subsequent analysis.

### Assessment of runs of homozygosity

In this study, we used the PLINK v1.9 to detect ROH across cattle genomes [56]. The specific parameters were set as follows: 50 SNPs sliding window was used to detect homozygous segments in each individual, and the sliding window allowed no more than 1 heterozygote. Several parameters of defining ROH are involved: (i) the minimum length was 500 kb; (ii) the proportion of homozygous overlap window was 0.05; (iii) the minimum number of consecutive SNPs included in an ROH was 100; (iv) the minimum SNP density was set to 50 kb/SNP; (v) the maximum gap between continuous homozygous SNPs was 100 kb; (vi) a maximum of two SNPs with missing genotypes and up to one heterozygous genotype were allowed in an ROH. ROH with different sizes were divided into three classes: (A) Small (500 kb to 1 Mb), (B) Medium (1 Mb to 5 Mb), and (C) Large (> 5 Mb), as described in a previous study [23].

### Correction test between ROH and production traits

Association analysis was performed between ROH and four production traits (NMW, ADG, CW, and LW). Prior to inclusion in the analysis, all phenotypes were adjusted using the general linear model (farm, year and sex are the fixed effects and weight before fattening and fattening days were covariates). Then we considered the residuals as the adjusted phenotype for later analysis. In this study, we first estimated the relationship between the total length of ROH for each individual and the production traits (NMW, CW, ADG, and LW) using the Pearson correction test.

Then, we divided the population into two subgroups with extreme phenotypes: the high 300 group and the low 300 groups. Analysis of consensus ROH was performed using PLINK with –homozyg-group option. We defined consensus ROH as segments of overlapping ROH that had a minimum of five SNPs. Statistical significance for the proportion of individuals with ROH differed between the high and low groups were assessed using Fisher’s exact test based on a 2 × 2 table for each ROH. The UCSC Genome Browser (https://genome.ucsc.edu/) was used to retrieve genes within genomic regions of interest based on bovine genome assembly (UMD 3.1).

### Association analysis of significant ROH

The set of significant ROH regions identified in the previous step was subsequently analyzed using the following linear mixed model:

$$\mathrm y=\mathrm{Xb}+\mathrm{Zu}+\mathrm e$$

where **y** is the vector of phenotypes for the studied traits; **b** is the vector of fixed effects including ROH, farm, year, sex, weight before fattening and fattening days. ROH that reach the significant level in the Fisher’s exact tests, are regarded as a binary variable (ROH presence/ROH absence). **X** and **Z** are the design matrices relating phenotypic traits to fixed and random effects, respectively; **u** is the vector of additive genetic effects and e is the vector of residual effects. The effects **u** and **e** were distributed as u ~ *N* (0, **G**${\sigma }_{g}^{2}$) and e ~ *N* (0, **R**${\sigma }_{e}^{2}$), where ${\sigma }_{g}^{2}$and ${\sigma }_{e}^{2}$are the additive genetic and residual variances, respectively, **G** is the additive genomic relationship matrix, which was constructed by synbreed packages based on SNPs [57, 58]. **R** is an identity matrix. The associations between each ROH region and phenotypic traits were evaluated using linear mixed model analysis in ASReml v3.0 [59] with a statistical significance level (P-value < 0.05). Linkage disequilibrium between SNPs around the target regions was estimated and visualized using Haploview v4.3.

## Supplementary Information

Additional file 1:

Additional file 2:

Additional file 3:

Additional file 4:

Additional file 5:

Additional file 6:

Additional file 7:

Additional file 8:

## Data Availability

Datasets are available from the Dryad Digital Repository (doi:10.5061/dryad.4qc06)
