# Supplementary figures and images for: Runs of homozygosity analysis reveals consensus homozygous regions affecting production traits in Chinese Simmental beef cattle
Source: BMC Genomics. 2021 Sep 21;22:678. doi: 10.1186/s12864-021-07992-6 (PMC8454143; doi:10.1186/s12864-021-07992-6)

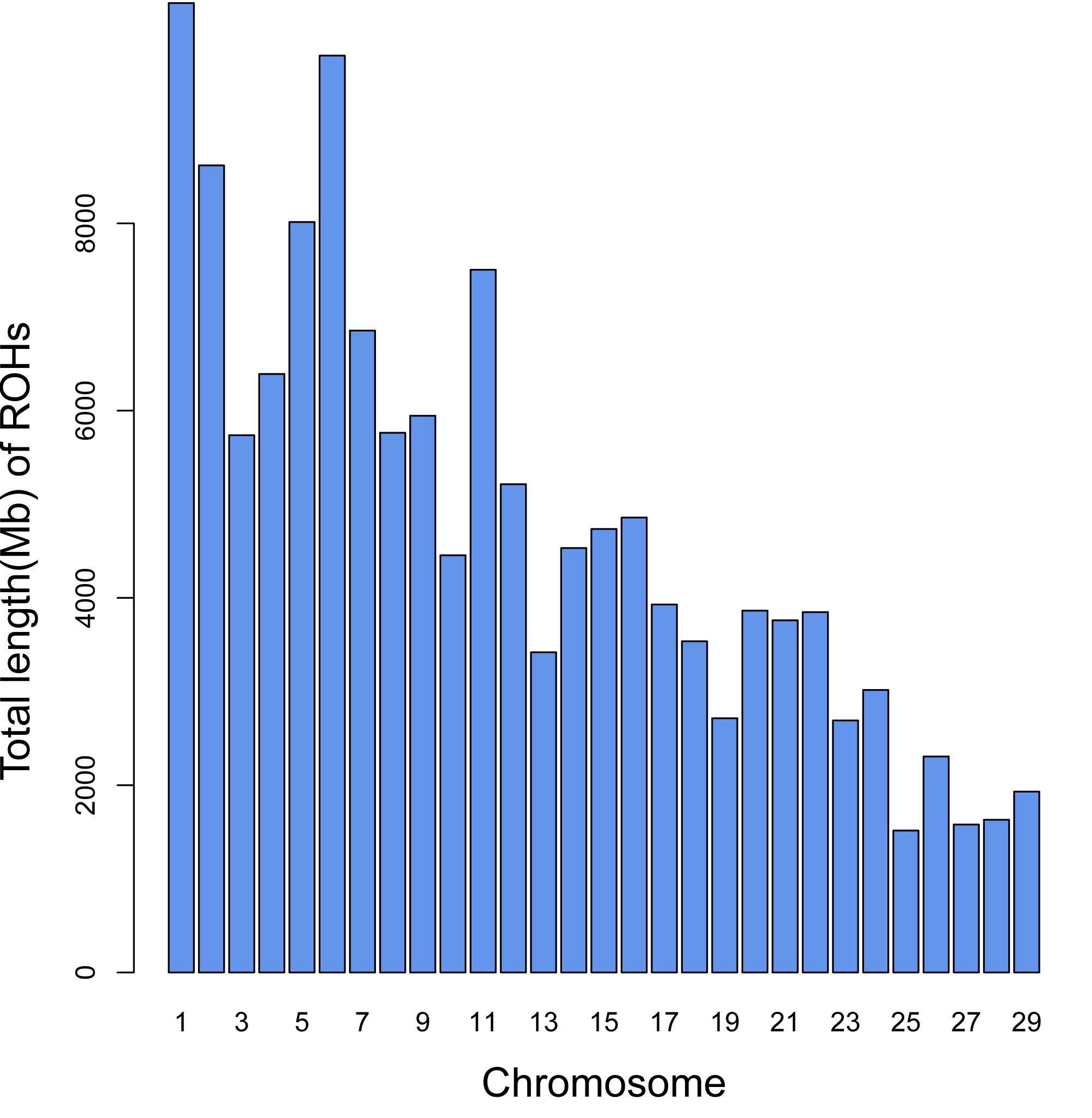

Supplement: Supplementary file 4 — Additional file 4: [file 12864_2021_7992_MOESM4_ESM.tif]

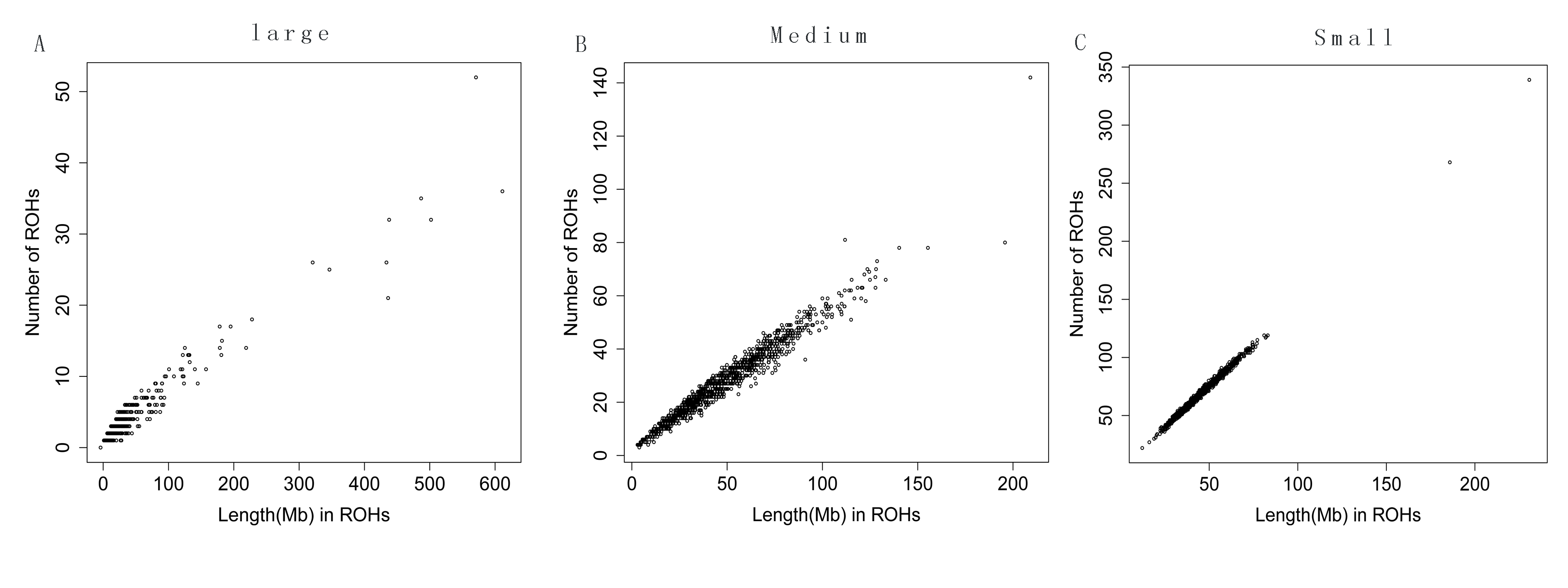

Supplement: Supplementary file 5 — Additional file 5: [file 12864_2021_7992_MOESM5_ESM.tif]

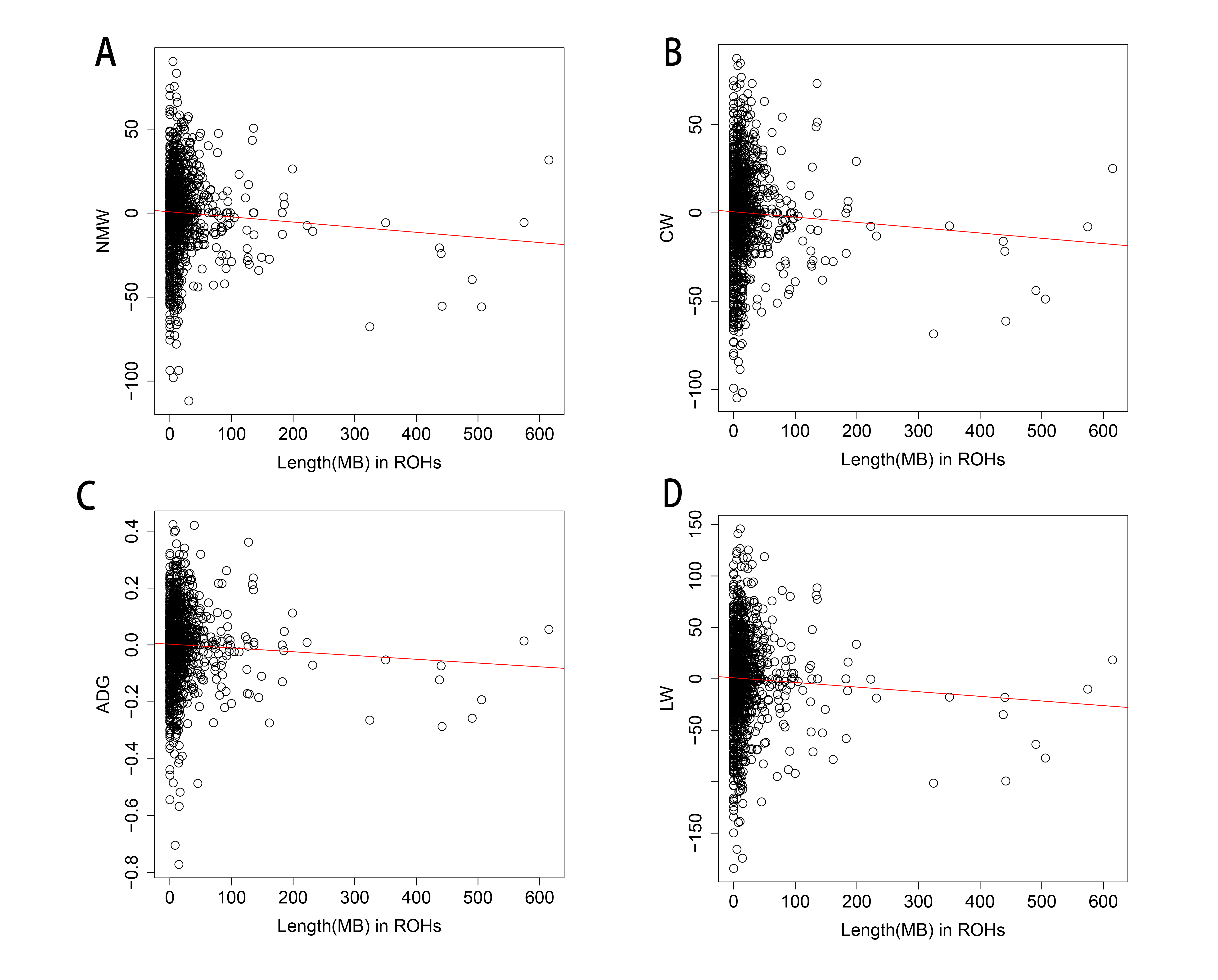

Supplement: Supplementary file 6 — Additional file 6: [file 12864_2021_7992_MOESM6_ESM.tif]

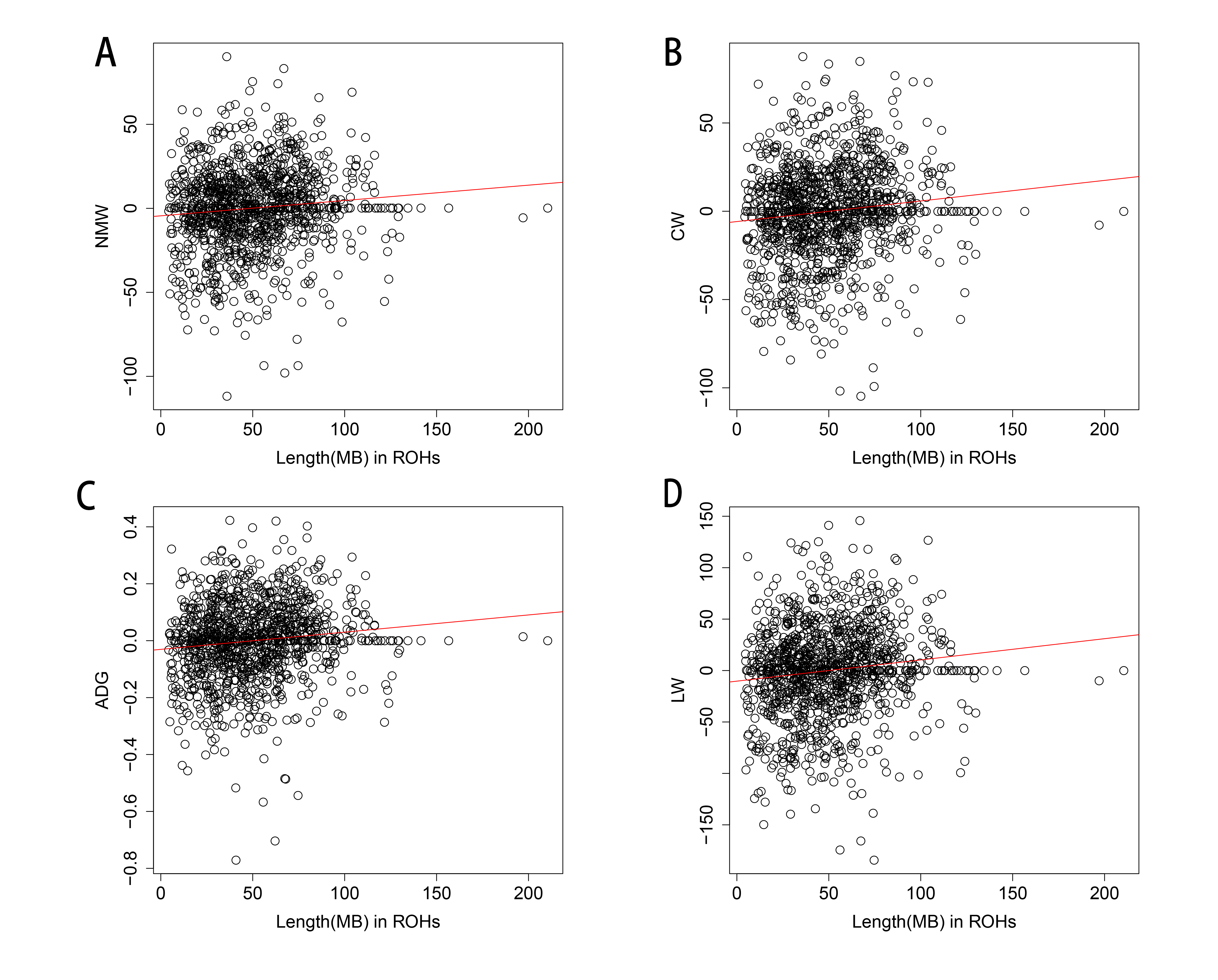

Supplement: Supplementary file 7 — Additional file 7: [file 12864_2021_7992_MOESM7_ESM.tif]

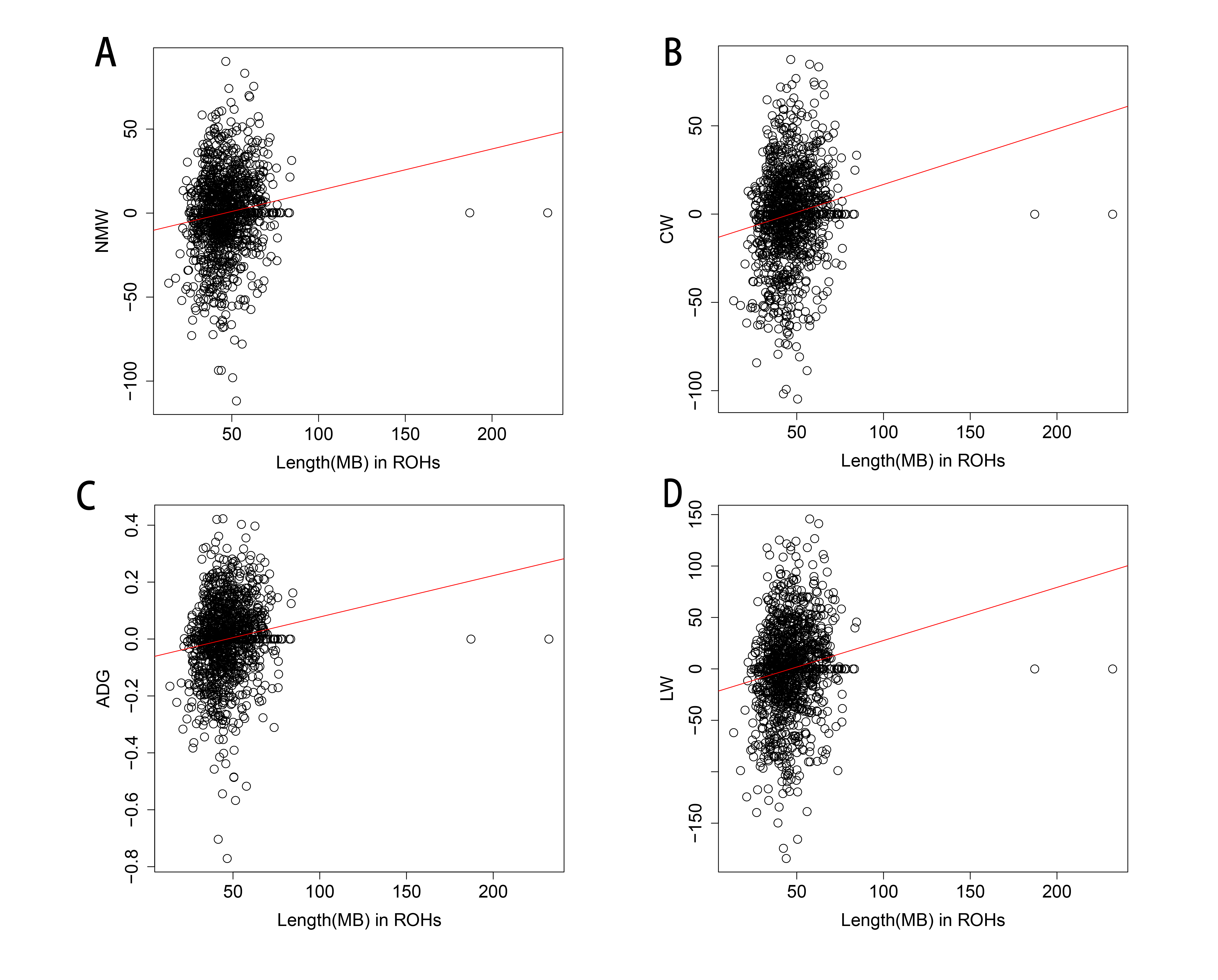

Supplement: Supplementary file 8 — Additional file 8: [file 12864_2021_7992_MOESM8_ESM.tif]
